# Research Ethics Boards: Reply from Trudo Lemmens and Carl Elliott

**DOI:** 10.1371/journal.pmed.0030471

**Published:** 2006-10-31

**Authors:** Trudo Lemmens, Carl Elliott

Our basic argument is this: for-profit research ethics boards are in a client–provider relationship with study sponsors; this relationship creates a conflict of interest; and this conflict of interest is particularly dangerous under a weak regulatory system which does not prevent forum shopping and allows market criteria to influence committee selection. We use various examples of serious controversies to support our claim that there are flaws in the system. This seems more appropriate than using one or two “good examples” to argue that the system works just fine. The claim by two for-profit research ethics boards that they manage to perform admirably despite this conflict of interest would not undermine our argument, even if independent evidence were given for the claim. (None is given here, of course.) Academic committees are certainly also affected by conflicts of interest. But that doesn't remove the conflict in for-profit review. The widely lauded system of accreditation is in our opinion a soft self-regulatory mechanism which does not fill the regulatory loopholes.

Lestou, Ondrusek, and Blajchman [1] are members of IRB Services, the for-profit research ethics committee that approved the controversial immunosuppressant study conducted at an Anapharm research unit in Montreal. As we noted in our article, which referred to a Bloomberg report [2], a number of subjects in that trial were infected with tuberculosis. Commentators (Steven Miles, and one of us—Lemmens) interviewed by Bloomberg criticized IRB Services for some aspects of its review, including for not imposing basic tuberculosis screening of participants and for approving a backloaded payment structure intended to keep people in the trial [2]. It is worth noting that Anapharm used at least one other prominent Canadian for-profit committee in the past [3], raising concerns about forum shopping.

We referred to WIRB in our article because WIRB is frequently lauded as setting the highest research ethics standards. Thus it seems particularly relevant that even WIRB has had its share of problems. Our reference to the sheer number of reviews conducted by WIRB reflects our concern about the power of one commercial entity in the exercise of what we consider a fundamental public function. WIRB has become a state within the state when it comes to the protection of human subjects. Particularly in the absence of strict regulatory control, it is worth asking whether it is healthy to concentrate so much power over the protection of human subjects in the hands of one commercial enterprise.

Shamoo and Woeckner [4] rightly point to the difficulty of gaining empirical evidence on research ethics boards. One of us conducted a survey of for-profit research ethics boards in 1997 [5]. While several committees collaborated well with this survey (including WIRB and IRB Services), much information remained hidden behind a veil of corporate secrecy. Regulatory agencies in Canada and the United States simply accept this secrecy. Some regulators even rely on the goodwill of research ethics boards to determine regulatory initiatives. When the US Food and Drug Administration looked into the issue of “IRB shopping,” for example, it invited comments, received them primarily from those in the industry, and simply accepted the claim that shopping for research ethics boards was not a serious concern [6].

The idea of regional committees is indeed not new, as Shamoo and Woeckner indicate. They cite as first source an article of 1995. However, research ethics committees with exclusive territorial jurisdiction have existed in several European countries since the late 1980s to early 1990s (e.g., France, Switzerland, and Denmark). They have since been introduced in many more countries. Emanuel suggests that we have failed to make positive contributions to the debate on the organization of research ethics boards and contrasts that with his 2004 proposal for regional review boards. In fact, one of us (Lemmens) has argued since 1996 for improvements to the regulatory structure of research ethics boards, including the introduction of regional committees, in scholarly journals [5,7–11], at international conferences, and in a report for the Council of Europe [9]. Excerpts of one of these articles [7] were reprinted in a text book on research ethics edited by Emanuel and others [12]. We are pleased to know that Emanuel has come to agree.
